# Commentary on: iCare Technique of Dissolving Ellanse M Nodules Using Collagenase: A Case Series and Experimental Study

**DOI:** 10.1111/jocd.70331

**Published:** 2025-07-16

**Authors:** Airá Novello Vilar, Vitoria Azulay, Arthur Cesar Farah Ferreira, Estevão Vargas, Rubem David Azulay, Vitor Azulay, Rosemarie Mazzuco

**Affiliations:** ^1^ Institute of Dermatology Professor Rubem David Azulay Santa Casa de Misericórdia Do Rio de Janeiro Rio de Janeiro Brazil; ^2^ Federal University Fronteira Sul Rio Grande do Sul Brazil; ^3^ Private Clinics Rio Grande do Sul Brazil

**Keywords:** collagen, collagen hydrolysate, poly‐L‐lactic acid

Dear Editor,

We would like to appreciate the effort that went into your manuscript in order to treat complications of injectable biostimulators. Therefore, as more patients seek this type of treatment, it is really important to ensure patient's safety not only during the procedure, but also managing its complication. Your work contributes to future studies and instigates a deeper understanding of managing biostimulator's complications.

While we acknowledge your successful study with Collagenase, it is important to highlight some concerns regarding its use.

Collagenase was the first injectable approved by the US Food and Drug Administration (FDA) for cellulite's treatment. The product used was collagenase clostridium histolyticum (Qwo^−^ by Endo International) which mechanism of action involves the hydrolysis of types I and III collagen, disrupting its septae [1, 2]. Furthermore, it is important to highlight that the vascular wall is composed of three distinct histological layers: intima, media, and adventitia. The adventitia, the outermost layer, is rich in type I collagen, guaranteeing the mechanical integrity of small and medium‐caliber vessels. This layer functions as a support mesh that protects the vessels from rupture due to mechanical forces and enzymatic degradation. In this sense, Qwo can cause blood vessel leakage (as it hydrolyses type I collagen) and, consequently, significant bruising [3, 4]. The risk of extensive hematomas, deep hemorrhages, and disorganization of tissue architecture arises from its direct action on the collagen matrix that supports vascular elements.

Its use proposed experimentally for the management of persistent nodules resulting from dermal fillers or biostimulators requires extreme caution when applied to anatomically complex areas such as the face [5].

The chronic inflammatory processes frequently associated with filler nodules—with the presence of fibrosis, reactive neovascularization, and aberrant remodeling of the matrix—already compromise local vascular stability. The introduction of collagenase into such an environment can potentiate matrix degradation and precipitate events of intense subcutaneous hemorrhage, formation of expansive hematomas, skin necrosis secondary to compression, and persistent discoloration due to hemosiderin deposits. Furthermore, patients with a history of hypersensitivity to collagenase or with coagulation disorders should avoid this medication in order to prevent anaphylaxis or massive bleeding [4].

Endo manufacturer attempted to mitigate these adverse effects through the APHRODITE study, which began in June 2022, but the results were not consistent enough to alleviate market concerns. For this reason, it was withdrawn from the market in December 2022. The decision was prompted by significant concerns regarding the extent and variability of bruising after initial treatment, as well as the risk of prolonged skin discoloration.

This pathophysiology explains the adverse events documented in the subcutaneous use of Qwo for cellulites, where significant bruising has occurred due to degradation of perivascular fibrous septa. In the face, where space is limited and vascular density is high, such events may be more severe and even fatal.

## Author Contributions

A.N.V. performed the research, designed the research study, and wrote the paper. V.A. performed the research, designed the research study, and wrote the paper. A.C.F.F. wrote the paper and analyzed the data. E.V. wrote the paper and analyzed the data. R.D.A. contributed essential tools and analyzed the data. V.A. contributed essential tools and analyzed the data. R.M. contributed essential tools and analyzed the data. All authors have read and approved the final version of the manuscript.

## Ethics Statement

The study was approved by the ethical review board.

## Conflicts of Interest

The authors declare no conflicts of interest.

## Data Availability

All data generated or analyzed during this study are included in this article. Further enquiries can be directed to the corresponding author.
